# A 3-Dimensional Bioprinted Decellularized Umbilical Cord Matrix Patch for Enhanced Storage and Delivery of Extracellular Vesicles in Diabetic Wound Healing

**DOI:** 10.34133/research.1246

**Published:** 2026-04-22

**Authors:** Ying Zhang, Hailan Liao, Xinyi Wei, Xiaojuan Zhu, Yingqi Zhou, Meixian Jin, Bo Zhao, Fen Yao, Danlei Wu, Yuan Wei, Shuqin Zhou, Qing Peng

**Affiliations:** ^1^ Department of Central Laboratory, The Second Affiliated Hospital, School of Medicine, The Chinese University of Hong Kong, Shenzhen and Longgang District People’s Hospital of Shenzhen, Shenzhen 518172, China.; ^2^ Department of Ophthalmology, The Second Affiliated Hospital, School of Medicine, The Chinese University of Hong Kong, Shenzhen and Longgang District People’s Hospital of Shenzhen, Shenzhen 518172, China.; ^3^ Department of Pharmacology, Shantou University Medical College, Shantou 515041, China.; ^4^ Department of Anesthesiology, First People’s Hospital of Kashi, Kashi 844000, China.; ^5^ Department of Anesthesiology, The Second Affiliated Hospital, School of Medicine, The Chinese University of Hong Kong, Shenzhen and Longgang District People’s Hospital of Shenzhen, Shenzhen 518172, China.; ^6^ Department of Basic Medicine, Changzhi Medical College, Changzhi 046000, China.

## Abstract

Extracellular vesicles (EVs) are promising cell-free therapeutics for diabetic wound healing due to their immunomodulatory and proangiogenic properties. Nonetheless, challenges in ensuring long-term stability and achieving targeted delivery continue to impede clinical translation. Herein, we developed a 3-dimensional bioprinted methacrylated decellularized umbilical cord matrix (MDUM) patch enabling the sustained delivery of telomerase-immortalized umbilical cord mesenchymal stem cell-derived EVs (TMSC-EVs). TMSC-EVs encapsulated in MDUM maintained their structural integrity and biological functionality for more than 30 d under 4 °C storage, outperforming those encapsulated in gelatin methacryloyl (*P* < 0.01). In a diabetic murine wound model, our data demonstrated that MDUM could enhance the retention and delivery of TMSC-EVs and further augment the therapeutic effects for diabetic wound healing as revealed by attenuating proinflammatory cytokine levels, enhancing neovascularization, and accelerating collagen deposition. This study pioneers the integration of biomaterial engineering with immortalized cell-derived EVs, establishing a translatable platform for regenerative therapies in chronic wound management.

## Introduction

Extracellular vesicles (EVs) are heterogeneous, lipid bilayer-delimited nanoparticles constitutively secreted by most cell types, where they serve as crucial messengers in intercellular communication [1,2]. Their functionality arises from transferring various bioactive molecules, including proteins, nucleic acids, lipids, and metabolites, to recipient cells, thereby influencing multiple physiological and pathological processes [3]. A growing body of research highlights that EVs are fundamental to maintaining cellular homeostasis and are pivotal in controlling key processes like angiogenesis, immune response, extracellular matrix (ECM) remodeling, and cellular differentiation [4–7]. These intrinsic properties, including biocompatibility, low immunogenicity, and stability, establish EVs as promising natural therapeutic agents and drug delivery systems in regenerative medicine.

Mesenchymal stem cell-derived extracellular vesicles (MSC-EVs) are nanosized (typically 30 to 150 nm in diameter), membrane-bound particles released by mesenchymal stem cells (MSCs) [8]. They carry regenerative cargo from their parent cells, including proangiogenic microRNAs (miRNAs), anti-inflammatory cytokines, and ECM-remodeling factors [9]. Consequently, MSC-EVs have attracted considerable attention as potential therapeutic agents for wound healing [10]. Compared to EVs derived from fibroblasts or immune cells, MSC-EVs offer advantages such as lower immunogenicity [11,12], higher production yield from scalable MSC cultures, and a well-established safety profile [13,14]. Research demonstrates that MSC-EVs accelerate skin wound repair in both diabetic and nondiabetic settings by modulating key processes, including the inflammatory response, angiogenesis, and ECM reorganization [15]. These vesicles contribute to all phases of the highly coordinated wound healing cascade, which encompasses hemostasis, inflammation, proliferation, and tissue remodeling [16].

Although EV-based therapies hold therapeutic promises, their clinical application encounters numerous challenges. A major limitation is their poor bioavailability and rapid clearance following administration. Conventional applications, such as subcutaneous injections around the wound, can cause secondary tissue damage; topical application as a wound dressing often leads to rapid clearance of bioactive cargo [17]; and systemic intravenous administration results in substantial sequestration by the mononuclear phagocyte system, with less than 1% of EVs remaining in circulation 24 h post-injection [18,19]. Furthermore, local administration is hampered by the rapid drainage of EVs from the target site into surrounding capillaries. Repeated dosing, while potentially necessary to maintain therapeutic levels, may disrupt the natural healing cascade by a persistent inflammatory stimulus. The stability of EVs can also be compromised by pathological microenvironmental factors prevalent in chronic wounds, such as elevated reactive oxygen species (ROS), proteolytic enzymes, and fluctuations in pH and ionic strength [20]. Besides, clinical translation faces critical barriers, and EVs’ bioactivity rapidly degrades within 7 d under standard 4 °C refrigeration [21], mandating costly −80 °C storage that induces EV aggregation and cargo leakage during freeze–thaw cycles [22].

**Fig. 1.**
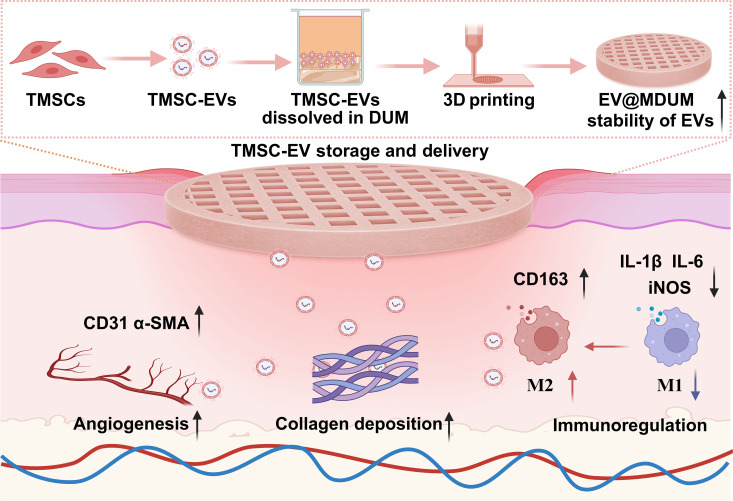
Scheme diagram of the fabrication of EV@MDUM and diabetic wound applications. The diagram is created in BioRender. EVs, extracellular vesicles; DUM, decellularized umbilical cord matrix; MDUM, methacrylated DUM; TMSC-EVs, telomerase-immortalized umbilical cord mesenchymal stem cell-derived extracellular vehicles; α-SMA, α-smooth muscle actin; 3D, 3-dimensional.

Hydrogel-based delivery systems offer a promising solution by mimicking native ECM architecture to prolong EV retention and provide mechanical protection. However, synthetic hydrogels lack the innate bioactive cues essential for diabetic wound regeneration [23]. Among synthetic hydrogels, gelatin methacryloyl (GelMA) has emerged as one of the most widely used photo-crosslinkable biomaterials for 3-dimensional (3D) bioprinting-based EV delivery due to its good printability and tunable mechanical properties [16]. However, as a single-component gelatin-derived material, GelMA lacks the biochemical complexity and intrinsic bioactivity preserved in native ECM. Beyond synthetic hydrogels such as GelMA, hyaluronic acid (HA)-based systems and microneedle patches represent widely explored alternatives. However, HA-based systems suffer from mechanical weakness and susceptibility to enzymatic degradation [24]. Moreover, HA lacks the tissue-specific bioactive complexity—such as collagens and endogenous growth factors—found in native ECM. Microneedles are inherently limited in drug loading capacity due to their microscale dimensions [25]. Additionally, microneedle applications may elevate the risk of deep infections in open diabetic wounds, which already have a high inherent susceptibility due to the compromised skin barrier [26]. Decellularized umbilical cord matrix (DUM) uniquely overcome these limitations; they are derived from surgical waste, process more immature collagen than adult tissues, and preserve tissue-specific biomolecules such as collagen III/IV, HA, and endogenous vascular endothelial growth factor that mirror skin ECM composition and synergize with EV cargo [27–29]. Comprising human-derived decellularized tissue, DUM exhibits inherently low immunogenicity, making it convenient for clinical translation. To harness this potential, we functionalized DUM with methacrylate (MA) groups to achieve photo-crosslinking, which allows rapid gelation and facilitates 3D printing. By leveraging this adaptability, we engineered a 3D-bioprinted patch from the methacrylated DUM (MDUM) as a prefabricated platform for enhancing diabetic wound healing (Fig. 1). This platform leverages DUM’s preserved native components to create shelters that shield EVs from enzymatic degradation, while precision bioprinting enables the spatiotemporal control of EV distribution within porous microfilaments, minimizing macrophage clearance and extending localized cargo release. Crucially, we employed telomerase-immortalized MSC-EVs (TMSC-EVs) to ensure batch-to-batch consistency, overcoming donor variability inherent to primary cell sources [30]. By demonstrating MDUM’s capacity to preserve EV functionality during logistics-friendly storage and potentiate wound closure in diabetic models, this work bridges personalized regenerative precision with scalable clinical deployment for diabetic wound management.

## Results

### Fabrication and characterization of MDUM

**Fig. 2.**
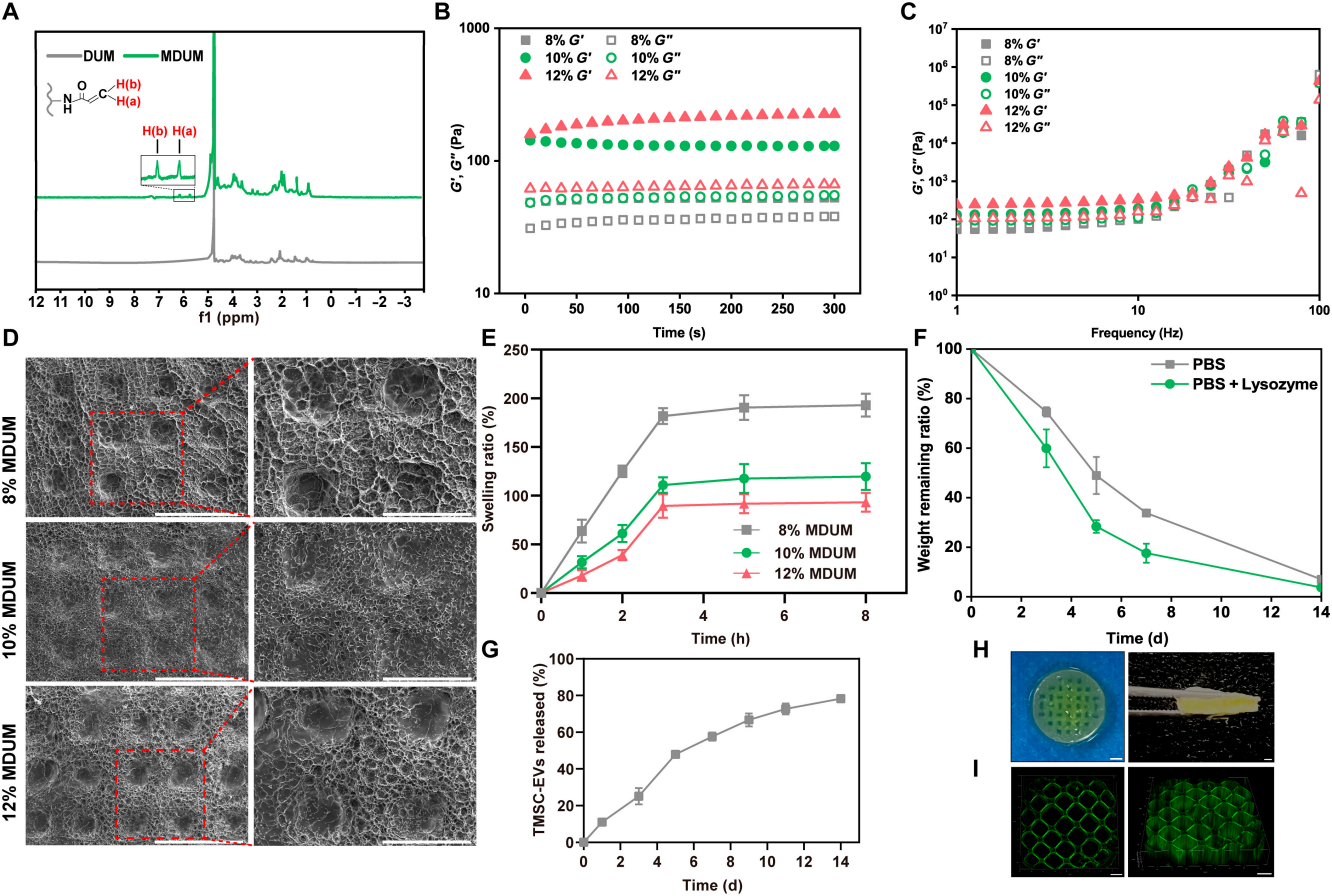
Synthesis and characterization of MDUM. (A) ^1^H NMR spectra of DUM and MDUM. (B) Time sweep and (C) frequency sweep rheological measurements of MDUM hydrogels at concentrations of 8%, 10%, and 12%, showing storage modulus (*G*′) and loss modulus (*G*″). (D) Scanning electron microscopy images showing the porous microstructures of 8%, 10%, and 12% MDUM hydrogels. Scale bar, 1 mm. (E) Time-dependent swelling ratio of MDUM hydrogels with different concentrations. (F) In vitro degradation behavior of 12% MDUM, evaluated by monitoring the weight remaining in PBS with or without lysozyme. (G) TMSC-EV release assay from EV@MDUM. (H) Photograph of 3D-bioprinted MDUM exhibiting a grid-like structure. Scale bar, 500 μm. (I) Representative 3D-bioprinted MDUM construct demonstrating high structural fidelity. Scale bar, 1 mm.

To expand the applicability of the DUM synthesized in our previous study [31], DUM was modified with MA groups and fabricated via a 3D-printing approach to obtain a photo-crosslinkable hydrogel, termed MDUM. ^1^H nuclear magnetic resonance spectroscopy revealed the appearance of characteristic double peaks at 5 to 6 ppm, confirming successful MA modification (Fig. 2A). The degree of MA substitution was calculated to be 88.68%. To develop the appropriate wound dressing, MDUM hydrogels with concentrations of 8%, 10%, and 12% were prepared, and their rheological properties were evaluated. Time and frequency sweep measurements (Fig. 2B and C) demonstrated stable storage modulus (*G*′) and loss modulus (*G*″) for all formulations, with *G*′ increasing as the MDUM concentration increased. These results indicate that MDUM maintains a stable gel state over a wide range of frequencies. To further characterize the physical properties of MDUM, scanning electron microscopy was employed to examine the pore architecture. Although freeze-drying may induce some morphological alterations, the 12% MDUM hydrogel exhibited the most compact and well-defined mesh structure (Fig. 2D). Swelling studies showed that MDUM rapidly absorbed water within the first 3 h and subsequently reached equilibrium (Fig. 2E). The swelling ratios of 8% MDUM (181.74 ± 8.11%) and 10% MDUM (110.93 ± 7.97%) were substantially higher than those of 12% MDUM (89.52 ± 12.05%). A lower swelling ratio can reduce mechanical stress at the wound site, thereby decreasing the risk of scar formation [32]. Accordingly, 12% MDUM was selected for subsequent experiments due to its high structural resolution and reduced swelling behavior.

The degradation behavior of MDUM was further assessed by monitoring weight loss in phosphate-buffered saline (PBS). The hydrogel mass decreased rapidly during the initial phase and gradually reached a plateau after 14 d (Fig. 2F), suggesting suitability for sustained and controlled release of encapsulated EVs. The release profile of EVs from MDUM was characterized in an in vitro study. TMSC-EVs were encapsulated within 12% MDUM, which was then immersed in PBS under gentle agitation. As shown in Fig. 2G, MDUM exhibited a sustained release profile. An initial burst release of approximately 25.10 ± 4.45% of the encapsulated EVs occurred within the first 3 d, likely due to the rapid diffusion of EVs located near the hydrogel surface. Subsequently, release continued in a controlled manner, reaching a cumulative release of 57.60 ± 2.26% by day 7 and 78.25 ± 0.66% by day 14. This sustained release behavior closely correlated with the in vitro degradation profile of MDUM (Fig. 2F). The sustained release over 2 weeks supports the role of MDUM as a depot for prolonged EV delivery. Finally, the printability of MDUM was evaluated by fabricating hydrogels with predesigned geometries. As shown in Fig. 2H and I, the 3D-bioprinted MDUM formed well-defined grid-like structures with excellent structural integrity, fidelity, and geometric resolution.

### MDUM enhances storage stability of TMSC-EVs

**Fig. 3.**
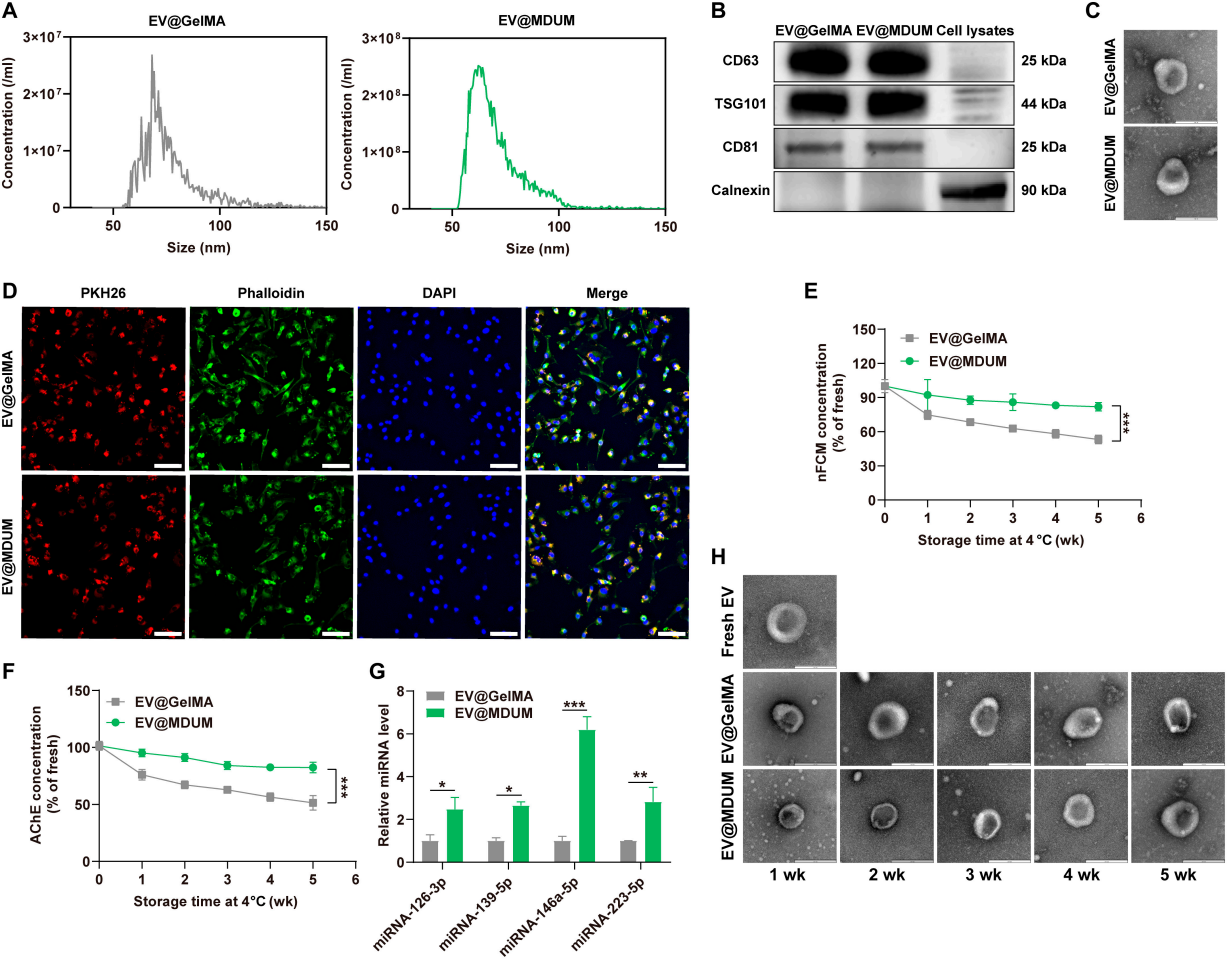
Characterization and storage stability of gelatin methacryloyl-incorporated TMSC-EVs (EV@GelMA) and EV@MDUM. (A) Size distribution of EVs determined by nanoflow cytometry (nFCM). (B) Western blot analysis of EV-specific markers (CD63, TSG101, and CD81) and the negative marker Calnexin in EV@GelMA, EV@MDUM, and cell lysates. (C) Transmission electron microscopy (TEM) images of EV@GelMA and EV@MDUM. Scale bar, 200 nm. (D) Cellular uptake of EV@GelMA and EV@MDUM by human umbilical vein endothelial cells (HUVECs). EVs were fluorescently labeled with PKH26 (red) and then incubated with HUVECs. The cytoskeleton was stained with phalloidin (green), and nuclei were counterstained with 4′,6-diamidino-2-phenylindole (DAPI) (blue). Uptake of labeled EVs was observed by confocal microscopy. Scale bar, 100 μm. (E) EV particle concentration measured by nFCM after lysis of hydrogels to release encapsulated EVs. (F) Acetylcholinesterase (AChE) concentration of EVs released from lysed hydrogels. (G) Relative quantification of EV-associated microRNA (miRNA) (miRNA-126-3p, miRNA-139-5p, miRNA-146a-5p, and miRNA-223-5p) after 5-wk storage at 4 °C, as determined by real-time quantitative PCR. (H) TEM images showing morphological changes of fresh EVs and EVs stored for 1 to 5 wk within GelMA or MDUM. Data are presented as mean ± SD. *P < 0.05, **P < 0.01, and ***P < 0.001. Statistical significance was analyzed using 1-way or 2-way analysis of variance (ANOVA), followed by post hoc tests. AChE, acetylcholinesterase.

TMSC-EVs were collected by cryogenic ultracentrifugation and subsequently encapsulated into MDUM to generate MDUM-incorporated TMSC-EVs (EV@MDUM). Using the same approach, GelMA-incorporated TMSC-EVs (EV@GelMA) were prepared. Nanoflow cytometry (nFCM) analysis showed that the average diameter of the EVs was approximately 70 nm (Fig. 3A). Western blot analysis confirmed that both EV@GelMA and EV@MDUM expressed the characteristic EV marker proteins CD63, TSG101, and CD81 and did not express the negative marker protein Calnexin, thereby confirming successful EV encapsulation and high preparation purity (Fig. 3B). To further examine the morphology and structural integrity of the EVs, transmission electron microscopy (TEM) was performed. As shown in Fig. 3C, EV@GelMA and EV@MDUM exhibited a typical cup-shaped morphology with intact membrane structures. Furthermore, in vitro cellular uptake assays demonstrated that TMSC-EVs encapsulated in both GelMA and MDUM were efficiently internalized by human umbilical vein endothelial cells (HUVECs) (Fig. 3D). Collectively, these results indicate that encapsulation within GelMA or MDUM does not alter the physicochemical properties of TMSC-EVs.

We hypothesized that MDUM could improve the storage stability of TMSC-EVs under refrigerated conditions. To quantify the EVs retained in the hydrogels after storage, the scaffolds were first lysed to release the encapsulated EVs for subsequent analyses. As shown in Fig. 3E, nFCM analysis revealed that the EV concentration in EV@GelMA decreased to 68.39 ± 2.76% after 2 weeks and further declined to 53.15 ± 3.76% after 5 weeks of storage, relative to freshly prepared EVs. In contrast, the EV concentration in EV@MDUM exhibited only a slight reduction to 87.67 ± 3.67% after 2 weeks and remained above 80% even after 5 weeks of storage.

In parallel, the stability of EV-associated components during storage at 4 °C was assessed. Acetylcholinesterase (AChE), a representative EV-associated protein, was used as an indicator of EV integrity, and changes in relative AChE activity closely mirrored the nFCM results. Specifically, the relative AChE activity in EV@GelMA declined to 67.25 ± 3.48% after 2 weeks and further decreased to below 55% after prolonged storage. By comparison, EV@MDUM preserved more than 80% of AChE activity for up to 5 weeks, showing no marked reduction over time (Fig. 3F).

We further evaluated the stability of miRNAs encapsulated in EV@GelMA and EV@MDUM using real-time quantitative polymerase chain reaction (qPCR). Previous studies have demonstrated that miRNAs including miRNA-126-3p, miRNA-139-5p, miRNA-146a-5p, and miRNA-223-5p are closely associated with the therapeutic effects of MSC-derived EVs [33–35]. Accordingly, these miRNAs were selected as representative indicators of EV miRNA stability. As shown in Fig. 3G, the relative miRNA levels of miRNA-126-3p, miRNA-139-5p, miRNA-146a-5p, and miRNA-223-5p in EV@MDUM were significantly higher than those in EV@GelMA after 5 weeks of storage. Additionally, TEM was used to examine potential structural changes in EVs during storage. As shown in Fig. 3H, no noticeable differences were observed in EV morphology, diameter, or membrane integrity between EV@GelMA and EV@MDUM after 5 weeks of storage. These findings are consistent with previous reports by Görgens et al. [36], which demonstrated that storage duration exerts minimal effects on EV morphology.

### MDUM enhances the therapeutic efficacy of TMSC-EVs in vitro

**Fig. 4.**
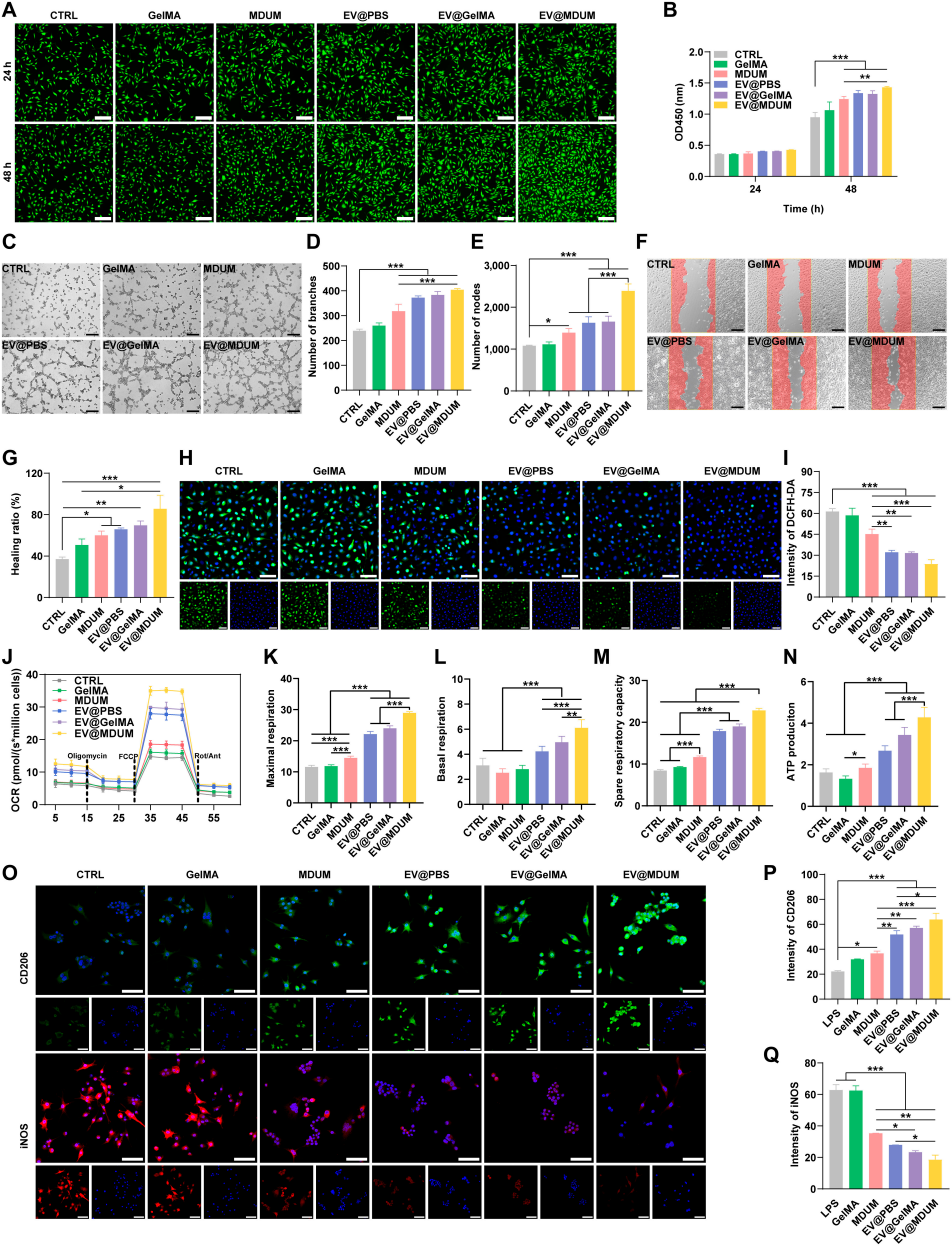
MDUM enhances the in vitro therapeutic effects of TMSC-EVs. (A) Representative calcein acetoxymethyl ester/propidium iodide staining images of HUVECs after 24 and 48 h of coculture with different formulations. Scale bar, 200 μm. CTRL, control group. (B) Quantitative Cell Counting Kit-8 analysis of HUVEC proliferation at 24 and 48 h. (C) Representative images of endothelial tube formation. Scale bar, 200 μm. (D and E) Quantification of node number and branch number, respectively. (F) Scratch wound healing assay of HUVEC migration. Scale bar, 200 μm. (G) Quantification of wound healing ratio. (H) Intracellular reactive oxygen species levels in phorbol 12-myristate 13-acetate-stimulated HUVECs detected by 2′,7′-dichlorodihydrofluorescein diacetate (DCFH-DA) staining. (I) Quantification of DCFH-DA fluorescence intensity. (J) Oxygen consumption rate (OCR) profiles following the sequential injection of oligomycin, carbonyl cyanide-4-(trifluoromethoxy)phenylhydrazone, and rotenone/antimycin A (Rot/Ant). (K to N) Quantitative analysis of maximal respiration (K), basal respiration (L), spare respiratory capacity (M), and adenosine triphosphate (ATP) production (N). (O) Immunofluorescence staining of CD206 and inducible nitric oxide synthase (iNOS) in lipopolysaccharide (LPS)-stimulated RAW264.7 macrophages. Scale bar, 50 μm. (P and Q) Quantification of CD206 (P) and iNOS (Q) fluorescence intensity. Data are presented as mean ± SD. Significance levels are denoted as follows: *P < 0.05, **P < 0.01, and ***P < 0.001. Statistical analysis involved 1-way or 2-way ANOVA, followed by post hoc tests.

To evaluate the therapeutic potential of EV@MDUM, we first examined their cytocompatibility and effects on endothelial proliferation. HUVECs were cocultured with GelMA, MDUM, EV@PBS, EV@GelMA, or EV@MDUM. Cytocompatibility was first assessed using calcein acetoxymethyl ester/propidium iodide staining and Cell Counting Kit-8 assays. Live/dead staining demonstrated high cell viability across all groups at both 24 and 48 h, confirming excellent cytocompatibility of the biomaterial scaffolds (Fig. 4A). Quantitative analysis of cell proliferation revealed that EV treatments promoted HUVEC growth, with a significant enhancement observed at 48 h. Notably, EV@MDUM induced the strongest proliferative response at this time point (Fig. 4B). MDUM alone also modestly promoted proliferation compared to the control and GelMA groups, indicating that the decellularized matrix itself possesses intrinsic pro-proliferative bioactivity. To further verify biosafety, hemocompatibility analysis demonstrated hemolysis rates below 2% for all materials (Fig. S1), satisfying accepted standards for blood-contacting biomaterials [37] and supporting subsequent biological evaluation.

Given that impaired angiogenesis is a defining feature of diabetic wound healing [38], we next investigated the proangiogenic effects of EV-loaded MDUM. In the tube formation assay, EV@MDUM markedly promoted the formation of dense, highly interconnected capillary-like networks on Matrigel. Quantitative analysis confirmed significant increases in both the number of nodes and branches compared to other groups (Fig. 4C to E). MDUM alone also slightly increased these parameters relative to the control. Similarly, in the scratch wound healing assay, EV@MDUM produced the most rapid wound closure (Fig. 4F and G). MDUM alone also accelerated endothelial cell migration compared to the control and GelMA groups. These results indicate that the MDUM matrix amplifies the intrinsic angiogenic activity of EVs.

Impaired vascularization in diabetic wounds is closely associated with excessive oxidative stress and mitochondrial metabolic dysfunction. We therefore investigated whether the enhanced angiogenic response induced by EV@MDUM was accompanied by regulation of intracellular ROS levels and mitochondrial oxidative phosphorylation. Using phorbol 12-myristate 13-acetate (PMA)-stimulated HUVECs as an oxidative stress model, intracellular ROS levels were assessed by 2′,7′-dichlorodihydrofluorescein diacetate staining. As shown in Fig. 4H and I, PMA stimulation induced substantial ROS accumulation. This was significantly mitigated by both MDUM alone and EV treatments, with the greatest reduction (strongest antioxidative effect) being observed with EV@MDUM. As mitochondrial dysfunction underlies oxidative stress-induced endothelial impairment, we assessed mitochondrial respiration. PMA stimulation markedly suppressed key oxygen consumption rate parameters, including basal and maximal respiration, spare respiratory capacity, and adenosine triphosphate production. EV@MDUM treatment induced the most significant recovery across all parameters (Fig. 4J to N), indicating effective metabolic regulation that promotes a shift toward an oxidative, energy-efficient state.

Because oxidative stress and mitochondrial dysfunction also sustain chronic inflammation by maintaining macrophages in a proinflammatory phenotype, we next evaluated whether MDUM and EV@MDUM could modulate macrophage polarization under inflammatory conditions. Figure 4O confirms that stimulation with lipopolysaccharide and interferon-gamma successfully induced M1 polarization in RAW264.7 macrophages, characterized by elevated inducible nitric oxide synthase (iNOS) expression, thereby establishing a robust inflammatory model. Under these conditions, both EV@MDUM and EV@GelMA significantly increased expression of the M2 marker CD206 while reducing iNOS levels. The effects of EV@MDUM were more pronounced than those of EV@PBS or MDUM alone (Fig. 4P and Q).

Collectively, these findings establish that EV@MDUM exerts strong, multifaceted pro-regenerative effects, including enhancing angiogenesis, alleviating oxidative stress, restoring mitochondrial function, and promoting reparative macrophage polarization.

### MDUM enhances the therapeutic efficacy of TMSC-EVs in vivo

**Fig. 5.**
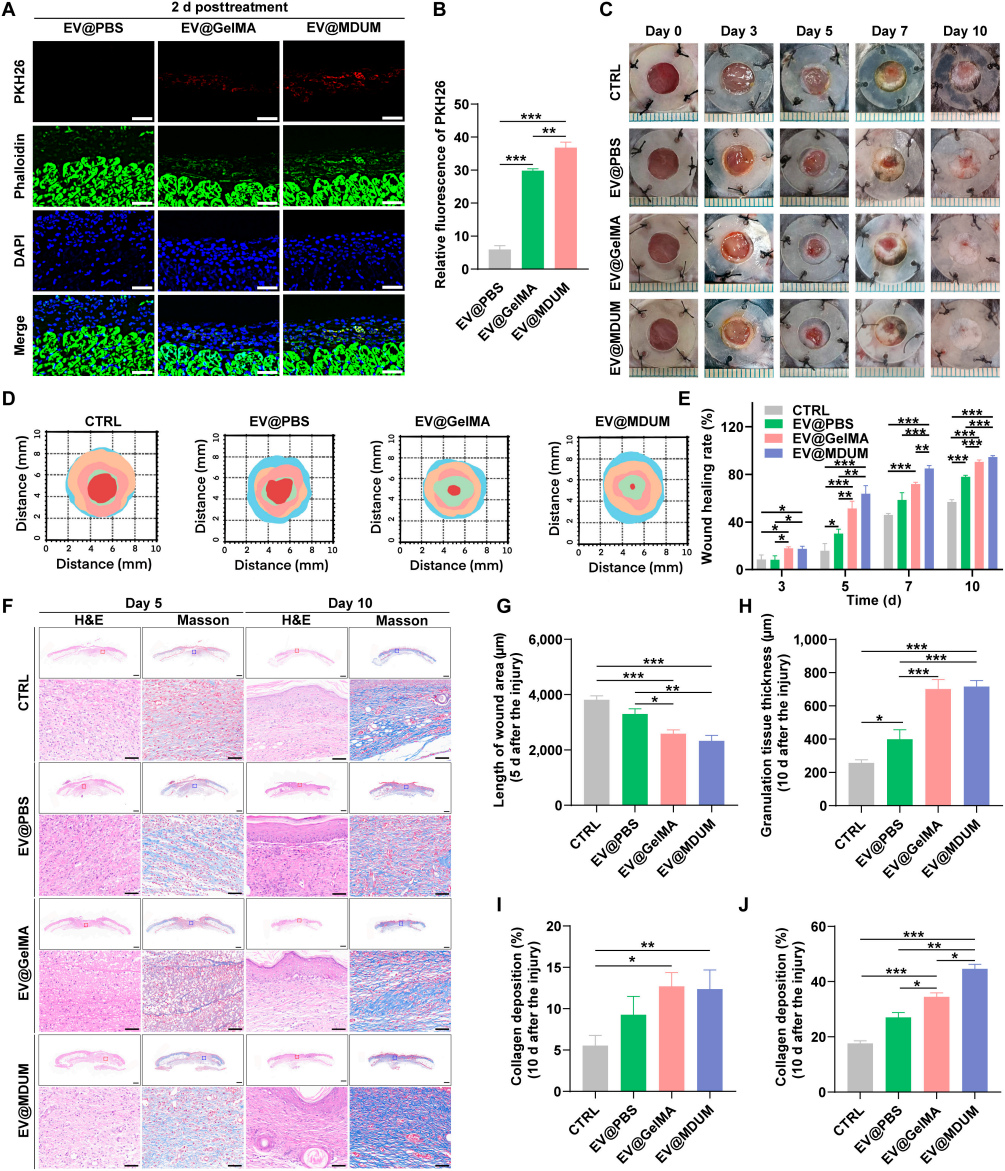
EV@MDUM promotes diabetic wound healing in vivo. (A) PKH26-labeled TMSC-EVs (red) formulated as EV@PBS, EV@GelMA, or EV@MDUM were topically applied to mice. Skin sections were harvested 2 d post-injection, stained with phalloidin (green), and analyzed by fluorescence microscopy. Scale bar, 50 μm. (B) Quantitative analysis of the PKH26-positive area. (C) Representative images showing wound healing progression in different treatment groups. (D) Representative gross images of wound healing progression in different treatment groups. (E) Quantification of wound closure rates over time. (F) Histological evaluation of wound tissue. Granulation tissue formation was assessed by hematoxylin and eosin (H&E) staining, and collagen deposition was evaluated by Masson’s trichrome staining on days 5 and 10 post-surgery. Scale bars, 500 μm (upper panels) and 50 μm (lower panels). (G) Quantification of wound length on day 5 post-surgery. (H) Quantification of granulation tissue thickness on day 10 post-surgery. (I and J) Quantification of collagen deposition on days 5 (I) and 10 (J). Data are presented as mean ± SD. Significance levels are denoted as follows: *P < 0.05, **P < 0.01, and ***P < 0.001. Statistical analysis involved 1-way or 2-way ANOVA, followed by post hoc tests.

To evaluate whether MDUM enhances the in vivo therapeutic efficacy of TMSC-EVs, we compared the biological performance of EV@PBS, EV@GelMA, and EV@MDUM in a diabetic wound model. We first assessed the in vivo retention of EVs. PKH26-labeled EVs formulated as EV@PBS, EV@GelMA, or EV@MDUM were topically applied to mice, followed by immunofluorescence analysis of skin sections 48 h after application. No detectable PKH26 fluorescence was observed in the EV@PBS group, indicating rapid clearance of free EVs. In contrast, clear PKH26 signals were retained in both the EV@GelMA and EV@MDUM groups, with the EV@MDUM group exhibiting the highest fluorescence intensity (Fig. 5A and B). These results demonstrate that MDUM significantly enhanced the local retention of EVs at the early stage (48 h post-application).

**Fig. 6.**
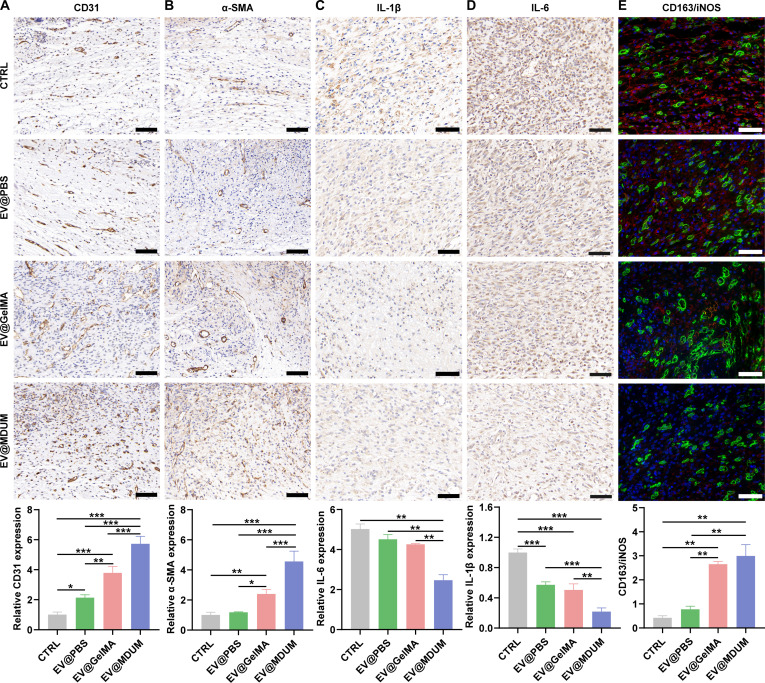
EV@MDUM enhances angiogenesis and modulates inflammation during wound healing. (A to D) Immunohistochemical staining of wound tissues from different treatment groups showing the expression of endothelial and inflammatory markers, including CD31 (A), α-SMA (B), IL-1β (C), and IL-6 (D). (E) Immunofluorescence staining for macrophage polarization, with CD163 used as a marker for M2 macrophages and iNOS as a marker for M1 macrophages. Scale bar, 50 μm. Data are presented as mean ± SD. Significance levels are denoted as follows: *P < 0.05, **P < 0.01, and ***P < 0.001. One-way ANOVA followed by post hoc tests was performed for statistical analysis.

Full-thickness dorsal wounds were created in diabetic mice and were treated topically with EV@PBS, EV@GelMA, or EV@MDUM. Wound healing progression was documented over time (Fig. 5C and D). Quantitative analysis revealed that wounds treated with EV@MDUM closed substantially faster than those in the other groups. By day 10 post-surgery, complete wound closure was achieved in the EV@MDUM group, whereas residual wound areas remained in the EV@PBS and EV@GelMA groups (Fig. 5E). Histological analyses were performed to evaluate tissue regeneration, including reepithelialization, granulation tissue formation, and collagen deposition (Fig. 5F). Hematoxylin and eosin (H&E) staining showed significantly reduced wound length and increased granulation tissue thickness in the EV@GelMA and EV@MDUM groups compared with the control and EV@PBS groups (Fig. 5G and H). Notably, Masson’s trichrome staining revealed markedly enhanced collagen deposition in the EV@MDUM group at both day 5 and day 10 post-surgery, exceeding that observed in all other groups (Fig. 5I and J). The proangiogenic effects of EV@MDUM were evaluated by immunohistochemical staining for CD31 and α-smooth muscle actin (α-SMA). The EV@MDUM group exhibited the largest CD31^+^ and α-SMA^+^ areas among all treatments, indicating enhanced neovascularization and vessel maturation during wound healing (Fig. 6A and B). Given the critical role of macrophages in regulating inflammatory responses during wound repair [39], we further examined inflammatory cytokine expression and macrophage polarization. Immunohistochemical analysis demonstrated significantly reduced expression of the proinflammatory cytokines interleukin-1β (IL-1β) and IL-6 in the EV@MDUM group (Fig. 6C and D). Moreover, EV@MDUM treatment resulted in the highest ratio of anti-inflammatory M2 macrophages (CD163^+^) to proinflammatory macrophages (iNOS^+^) at wound sites (Fig. 6E), indicating a pronounced shift toward M2 polarization.

Collectively, these results demonstrate that MDUM markedly enhances the therapeutic efficacy of TMSC-EVs in vivo by improving EV retention, accelerating wound closure, promoting angiogenesis, suppressing excessive inflammation, facilitating reepithelialization, and enhancing organized collagen deposition. To assess systemic biosafety, major organs including the heart, liver, spleen, lung, and kidney were harvested at the end of the animal study and subjected to H&E staining. No apparent histopathological abnormalities, inflammatory infiltration, or tissue damage were observed in the EV@MDUM, EV@GelMA, EV@PBS, or control groups, indicating that the treatments did not induce detectable systemic organ toxicity (Fig. S2).

## Discussion

The development of biomaterial-assisted EV delivery systems represents a promising strategy to overcome the translational bottlenecks of EV-based therapies in chronic diabetic wounds. Among various biomaterial platforms, decellularized ECM hydrogels have attracted increasing attention due to their ability to preserve native biochemical cues that support tissue regeneration and stabilize therapeutic cargos [40], while other decellularized ECM hydrogels, such as those derived from skin, cardiac, or adipose tissues, may similarly protect EVs through their preserved native components. Umbilical cord matrix offers distinctive advantages of cutaneous wound healing, exceptionally high type III collagen content mimicking fetal regenerative capacity, elevated HA and endogenous vascular endothelial growth factor, and low immunogenicity as surgical waste material [41]. Given that autologous umbilical cord tissue is unavailable for adult patients, DUM is considered an allogeneic biomaterial source. Its practical availability from donated tissue and low immunogenicity post-decellularization make allogeneic transplantation a viable and widely adoptable strategy for clinical translation. In this study, we demonstrated that 3D-bioprinted MDUM functions not only as a passive EV carrier but also as a scaffold that synergistically amplifies the therapeutic efficacy of TMSC-EVs. By integrating structural biomimicry, immune and metabolic regulation, and storage stability enhancement, this platform addresses several long-standing challenges in EV clinical deployment.

Unlike synthetic hydrogels such as GelMA, which primarily provide mechanical encapsulation and diffusion-controlled release, MDUM retains native ECM components derived from Wharton’s jelly, including collagen III/IV, glycosaminoglycans, and endogenous growth residues. These elements are increasingly recognized as critical regulators of cell–matrix crosstalk in wound healing [42]. Recent studies [43] highlight that decellularized matrices preserve tissue-specific biochemical cues capable of modulating angiogenesis, fibroblast activity, and immune cell behavior in ways that synthetic polymers cannot fully recapitulate. Consistent with this paradigm, MDUM exhibited superior EV retention, enhanced angiogenesis, and improved collagen organization in vivo compared with GelMA-based delivery, underscoring its advantage as a biologically instructive scaffold rather than a neutral depot.

Furthermore, the optimized 12% MDUM formulation demonstrated favorable mechanical stability, reduced swelling, and controlled degradation kinetics; these properties are essential for minimizing mechanical stress on fragile diabetic wound beds while enabling sustained EV release. This structural optimization likely contributed to the prolonged local bioavailability of EVs observed in vivo, addressing the rapid clearance that has limited the efficacy of topical or injectable EV therapies.

Beyond structural advantages, a key finding of this work is the ability of EV@MDUM to modulate immune and metabolic dysfunction, a hallmark of diabetic wound pathology. Chronic hyperglycemia induces mitochondrial impairment and excessive ROS production, which perpetuate endothelial dysfunction and lock macrophages in a proinflammatory (M1) phenotype. Emerging evidence indicates that restoring mitochondrial oxidative phosphorylation and redox balance is critical for effective wound resolution [44].

Our data demonstrate that EV@MDUM markedly restores mitochondrial respiratory capacity and attenuates oxidative stress in endothelial cells, suggesting improved metabolic homeostasis. These metabolic regulatory effects facilitate endothelial survival, migration, and angiogenic capacity under diabetic conditions. Notably, the enhanced antioxidant and metabolic effects observed with EV@MDUM exceeded those of EVs delivered through GelMA, implying that MDUM may provide a protective microenvironment that preserves EV cargo activity and possibly participates in redox buffering. In our study, a divergence was observed between ultrastructural and functional assessments of EV stability. While TEM showed preserved vesicular morphology during storage, nFCM and qPCR analyses revealed reductions in EV recovery and miRNA integrity. This morphology function dissociation likely arises from storage-associated molecular alterations, such as membrane permeability changes or cargo degradation [45], which are not detectable by conventional TEM imaging. The ECM-derived microenvironment provided by MDUM may stabilize EV membranes and limit cargo loss, thereby preserving functional integrity despite minimal ultrastructural differences.

Concurrently, EV@MDUM promoted macrophage phenotypic transition from a proinflammatory M1 state toward a reparative M2 phenotype both in vitro and in vivo. This shift is increasingly understood as a metabolically driven process, tightly coupled to mitochondrial function and ROS levels. EV-associated miRNAs such as miRNA-146a-5p and miRNA-223-5p have been reported to suppress nuclear factor kappa-light-chain enhancer of activated B cell signaling and inflammatory cytokine production [46,47], while MDUM-mediated ROS attenuation may further lower the activation threshold for M2 polarization. Together, these findings position EV@MDUM as a system capable of coordinating immune regulation and metabolic responses.

Compared with previously reported EV-loaded hydrogels, microneedle patches, or ECM-derived scaffolds, the MDUM platform offers several distinctive advantages. First, its human-derived decellularized matrix composition provides intrinsic bioactivity and low immunogenicity, facilitating clinical translation. Second, the 3D bioprinting strategy enables precise architectural control, which may be further exploited to tailor porosity and EV distribution for patient-specific wounds. Third, the use of hTERT-immortalized MSCs ensures batch-to-batch consistency of EVs, addressing donor variability.

Importantly, MDUM markedly improved EV storage stability at 4 °C for over 30 d, maintaining protein activity and miRNA integrity without the need for cryopreservation. This feature distinguishes EV@MDUM from most reported systems that still rely on −80 °C storage or lyophilization, both of which increase cost and risk of cargo degradation [22]. From a translational perspective, this cold-chain-friendly stability represents a substantial step toward “off-the-shelf” EV therapeutics. We speculate that the superior EV storage stability observed in MDUM likely arises from multiple synergistic protective mechanisms. First, the 3D network structure of the matrix gel can physically encapsulate EVs, reducing aggregation or degradation, and may to some extent buffer mechanical stress or enzymatic hydrolysis. Second, endogenous HA (negative charge) and heparan sulfate proteoglycans may interact with the EVs’ membrane surface through charge or intermolecular interactions, helping to maintain membrane integrity [48,49]. Further investigation is needed to elucidate the precise molecular interactions and validate these hypotheses.

Despite these promising results, several limitations warrant consideration. First, this study focused on 4 °C storage (the standard clinical condition) without evaluating room-temperature stability. While MDUM’s inherent antioxidant properties and dense ECM architecture may confer partial protection at 25 °C, systematic assessment at ambient and physiological temperatures is needed to define stability thresholds, degradation kinetics, and cargo integrity. Achieving truly cold-chain-independent EV therapeutics would substantially enhance global accessibility, particularly in resource-limited settings. Second, while mitochondrial respiration and ROS levels were assessed, the precise molecular pathways linking EV cargo, MDUM-derived cues, and metabolic regulation remain incompletely defined. Future transcriptomic or metabolomic profiling could elucidate these regulatory networks. Third, although the diabetic mouse model captures key aspects of impaired wound repair, it does not fully recapitulate the complexity of human chronic wounds, such as infection or ischemia. Evaluation in large-animal or infected wound models will be essential for clinical translation.

## Conclusion

This study introduces an innovative MDUM, serving as a robust platform for the prolonged storage and delivery of TMSC-EVs. MDUM effectively addresses the instability of EVs during cold-chain storage, maintaining over 80% bioactivity retention at 4 °C for 5 weeks. Furthermore, it synergizes with EVs to remodel the diabetic wound microenvironment, exerting anti-inflammatory, proangiogenic, and metabolic regulatory effects. By integrating biomaterial engineering with standardized EV production, this work establishes a translatable therapeutic strategy for chronic wounds, thereby addressing critical challenges in EV-based regenerative medicine.

## Materials and Methods

### Materials and reagents

Lithium phenyl-2,4,6-trimethylbenzoylphosphinate and GelMA were sourced from Yongqinquan Intelligent Equipment Co. Ltd. (Suzhou, China). The hTERT-MSCs utilized in this study were sourced from our prior research [30] and maintained in serum-free medium (TBD, Tianjin, China). HUVECs were sourced from Thermo Fisher Scientific (MA, USA) and maintained in serum-free medium (Thermo Fisher Scientific, MA, USA). For animal experiments, 6- to 8-week-old male C57BL/6 mice were sourced from the Animal Center of The Chinese University of Hong Kong, Shenzhen.

### Preparation and characterization of TMSC-EVs

To isolate TMSC-EVs, the method from our prior study was employed [30]. In summary, hTERT-MSCs were cultured until 80% confluence, after which the medium was replaced. Cell-free supernatants were collected after 48 h by subjecting the culture medium to 2 centrifugation cycles at 1,500*g* for 15 min each. EV isolation was then processed with ultrafiltration (100-kDa cutoff) and size exclusion chromatography (Exosupur column; ECHO Biotech). EV characterization included morphological analysis by TEM (Hitachi, Japan) and particle size measurement by nFCM. Identification of EVs was performed by Western blotting using anti-CD63, anti-TSG101, anti-CD81, and anti-Calnexin (1:1,000; Santa Cruz Biotechnology, CA) antibodies.

### AChE activity

AChE levels in EV samples were quantified with a commercial assay kit (Solarbio, Beijing, China). EVs (3 × 10^8^ particles) were distributed into a 96-well plate and combined with 50 μl of the working solution. After incubating for 30 min in the dark at 25 °C, absorbance was recorded at 410 nm with microplate readers. The enzyme concentration was measured from a kit-provided standard curve, with freshly isolated EVs serving as the control.

### Isolation of RNA and its subsequent analysis using qPCR

Total RNA was extracted using Trizol reagent. Prior to extraction, EV particle concentrations were quantified by nFCM and samples were diluted to identical particle numbers (3 × 10^8^ particles). Synthetic cel-miR-39-3p (5 fmol) was added as a spike-in control during RNA extraction. Complementary DNA (cDNA) synthesis was performed using the miRNA cDNA First Chain Synthesis Kit (Accurate Biology). qPCR was conducted using SYBR Green qPCR kit (Accurate Biology) with primers in Table S1. Relative expression was calculated by the 2^(−ΔΔCt)^ method using cel-miR-39-3p as reference.

### In vitro EV uptake by HUVECs

The red fluorescent dye PKH26 (Umibio, China) was used to label the purified EVs following the supplier’s protocols. Briefly, EV pellets were resuspended and incubated with 5 μM PKH26 in Diluent C for 10 min at 25 °C in the dark. The reaction was terminated by adding 10 ml of PBS, and unincorporated dye was removed by ultracentrifugation (20,000*g*, 15 min, 4 °C), with the final pellet resuspended in sterile PBS. For the in vitro uptake assay, HUVECs were seeded in confocal dishes at a density of 5 × 10^4^ cells/cm^2^ and grown to 80% confluency before being incubated with the PKH26-labeled EVs for 6 h at 37 °C. Post-incubation, cells were washed with ice-cold PBS, followed by fixing with 4% paraformaldehyde, permeabilizing with 0.1% Triton X-100, and counterstaining with fluorescein isothiocyanate–phalloidin and 4′,6-diamidino-2-phenylindole (Beyotime Biotechnology, China) to visualize F-actin and nuclei, respectively. Imaging was performed on a Leica DMi8 fluorescence microscope.

### In vivo EV uptake in skin tissue

In the in vivo uptake study, C57BL/6 mice received topical patch application of PKH26-labeled MSC-EVs (100 μg) in 50 μl of PBS into the diabetic wound area. The animals were euthanized 48 h later, and skin tissues from the patch application site were collected. Tissues were embedded in optimal-cutting-temperature compound, sectioned at 10-μm thickness, and fixed with 4% paraformaldehyde for 15 min. Subsequent staining with fluorescein isothiocyanate–phalloidin and 4′,6-diamidino-2-phenylindole was carried out before examination under the Leica DMi8 microscope to assess the distribution of PKH26 fluorescence in the skin layers.

### Preparation of DUM

Human umbilical cord tissues were obtained from The Second Affiliated Hospital, School of Medicine, The Chinese University of Hong Kong, Shenzhen, with written informed consent from donors. The study was approved by the Institutional Review Board of The Second Affiliated Hospital, The Chinese University of Hong Kong, Shenzhen (approval no. 2025131). ECM was prepared by decellularization according to a previously established protocol in our laboratory [31], which included standardized criteria for donor selection and DNA content quality control. To prepare the DUM bioink, 2 g of ECM lyophilized powder was dissolved in 50 ml of deionized water and 0.4 g of pepsin was added to achieve a pH value of 1.0. After complete dissolution, the pH was neutralized to 7.0 with 2 M NaOH. Glycidyl methacrylate (8 ml; Sigma-Aldrich) was then slowly added (0.5 ml/min) for methacrylation. The resulting product was dialyzed and lyophilized. The chemical structure and the degree of methacrylation of the DUM hydrogel were characterized by ^1^H nuclear magnetic resonance spectroscopy (Bruker, 500 MHz) after rehydrating lyophilized samples in D_2_O, with data analysis performed using MestReNova software according to the established method [50].

### Fabrication of MDUM

MDUM constructs were synthesized using a desktop bioprinter (EFL-BP-6800; Suzhou Intelligent Manufacturing Research Institute, Suzhou, China) with DUM bioink at varying concentrations (8%, 10%, and 12% wt %). Following deposition, the printed structures were crosslinked by exposure to 300 mW/cm^2^ ultraviolet light after adding lithium phenyl-2,4,6-trimethylbenzoylphosphinate as a photoinitiator. To eliminate the residual photoinitiator, the printed architecture was transferred to a 6-well cell culture plate and subjected to 3 washing cycles with PBS.

### Characterization of MDUM

Scanning electron microscopy (Hitachi SU8100, Japan) was used to analyze the microstructural morphology of MDUM across various DUM concentrations. Samples were sputter-coated with gold for 30 s before imaging. Rheological properties were assessed at 37 °C using a Kinexus Pro rotary rheometer (Malvern, UK) equipped with a 25-mm parallel-plate setup. *G*′ and *G*″ were assessed at a constant 1% strain across a frequency spectrum of 0.1 to 10 Hz. The swelling capacity was evaluated by immersing MDUM samples of known initial weight (*W*_0_) in 50 ml of PBS at pH 7.4. Once equilibrium swelling was achieved, surface PBS was eliminated, and the saturated weight (*W*_1_) was documented. The swelling ratio (SR) is determined using the formula SR = [(*W*_1_ − *W*_0_)/*W*_0_] × 100%. For degradation analysis, lyophilized 12% MDUM scaffolds (initial weight *W*_0_) were rehydrated in PBS for 24 h and subsequently incubated at 37 °C in PBS, both with and without 1,000 U/ml of lysozyme. Hydrogels were periodically lyophilized, and their dry weight (*W*_t_) was recorded. The percentage of remaining mass (RM) was determined as RM (%) = [(*W*_0_ − *W*_t_)/*W*_0_] × 100%. To measure the release curve of TMSC-EVs from EV@MDUM, EV@MDUM was incubated in 1 ml of PBS at 37 °C. The PBS was replaced daily, and the amount of EVs released into the supernatant was quantified by nFCM at each time point over 14 d.

### Biological evaluation of EV@MDUM

Cell viability: HUVECs were cultured for 1-, 3-, and 5-d intervals. Calcein acetoxymethyl ester/propidium iodide was used to detect cell viability and imaged with a Leica DMi8 fluorescence microscope, followed by Cell Counting Kit-8 assay.

Hemocompatibility: Mouse whole blood was collected in anticoagulant tubes and processed to obtain an erythrocyte suspension. The suspension was diluted with saline and incubated with patches for 4 h at 37 °C, followed by centrifugation. Controls included 0.1% Triton X-100 (positive) and saline (negative).

Tube formation assay: HUVECs (1 × 10^5^ cells) were plated onto Matrigel in 48-well plates and incubated in serum-free medium for 6 h. Tube formation was imaged with an inverted microscope (Primovert; ZEISS), and nodes/branches were quantified using ImageJ.

Cell migration assay: A scratch wound assay was performed on HUVEC monolayers. Cells were cultured in 6-well plates, and a scratch was made using a pipette tip. Migration was monitored at 24 and 48 h post-scratch.

Intracellular ROS scavenging: HUVECs were exposed to 1 μg/ml of PMA for 2 h to induce oxidative stress. ROS levels were measured using a 2′,7′-dichlorodihydrofluorescein diacetate probe and fluorescence microscopy.

Macrophage repolarization: RAW264.7 cells were polarized to the M1 phenotype using interferon-gamma and lipopolysaccharide and then treated with EV formulations to achieve macrophage repolarization. M1/M2 macrophage ratios were determined by staining for iNOS and CD206.

Oxygen consumption rate: HUVECs, at a concentration of 2 × 10^6^ cells/ml, were measured by resuspending them in MiR05 respiration buffer (0.5 ml per chamber) and placing them in the dual chambers of the Oroboros O2k system. The experiment was conducted at 37 °C with continuous stirring (750 rpm). Sequential substrate–uncoupler–inhibitor titration (SUIT protocol) was executed following the guidelines provided by the complete cell detection kit from Huawei, China. Oxygen consumption rate was recorded via polarographic sensors (DatLab v7.4).

### Animal model and experimental design

All animal procedures were performed in accordance with the guidelines of the Institutional Animal Care and Use Committee of The Second Affiliated Hospital, School of Medicine, The Chinese University of Hong Kong, Shenzhen (approval no. 20250123DW). A total of 40 healthy 6-week-old male C57BL/6 mice (weighing 20 to 25 g) were obtained from the hospital’s Experimental Animal Center. A diabetic mouse model was induced via intraperitoneal injection of streptozotocin, with diabetes defined as sustained blood glucose levels over 16.7 mmol/l for a minimum of 1 month. These diabetic mice were then randomly divided into 4 experimental groups (*n* = 10 per group): a control group (CTRL) and groups receiving TMSC-EVs suspended in PBS (EV@PBS), encapsulated in GelMA (EV@GelMA), or encapsulated in MDUM (EV@MDUM). For wound creation, mice were anesthetized by intraperitoneal injection of 0.3% phenobarbital sodium (0.1 ml per 10 g of body weight). A 6-mm full-thickness cutaneous wound was excised along the dorsal midline, and silicone splint rings were sutured around the wound margins to minimize contraction. For treatment, preformed hydrogel patches containing the respective formulations were gently placed to fully cover the wound bed immediately after injury. The patches adhered to the wound surface without additional fixation and remained in direct contact with the tissue throughout the treatment period. Wound healing was monitored by capturing photographs on days 0, 3, 5, 7, and 10 after wounding. The wound area was quantified by tracing the wound margin using ImageJ software.

For histological and immunohistochemical evaluation, wound tissues were harvested at predetermined time points. Specimens were fixed, embedded in paraffin, and sectioned into 4-μm-thick slices for staining. H&E staining was used to evaluate the general morphology, while Masson’s trichrome staining assessed collagen deposition. Neovascularization was assessed using immunohistochemical staining for CD31 and α-SMA, whereas inflammation was evaluated through IL-1β and IL-6 staining. The proportions of M1 (iNOS^+^) and M2 (CD206^+^) macrophages were quantified via immunofluorescence.

### Statistical analysis

Statistical analyses were performed using GraphPad Prism 8.0. Differences among groups were assessed by 1-way or 2-way analysis of variance (ANOVA), followed by Tukey’s post hoc test. Data are presented as mean ± standard deviation. Each data point represents the average of at least 3 independent experiments, each with 3 replicates. A *P* value of less than 0.05 was considered statistically significant (*P < 0.05, **P < 0.01, and ***P < 0.001).

## Data Availability

The data supporting this study’s findings are available within the article and its supplementary materials.
