# Supplementary material for: A 3-Dimensional Bioprinted Decellularized Umbilical Cord Matrix Patch for Enhanced Storage and Delivery of Extracellular Vesicles in Diabetic Wound Healing
Source: Research (Wash D C). 2026 Apr 22;9:1246. doi: 10.34133/research.1246 (PMC13100348; doi:10.34133/research.1246)
Supplement: Supplementary 1 — Figs. S1 and S2 Table S1 [file research.1246.f1.zip › Supplementary Materials.docx]

SUPPLEMENTARY MATERIALS

Figures S1 to S2


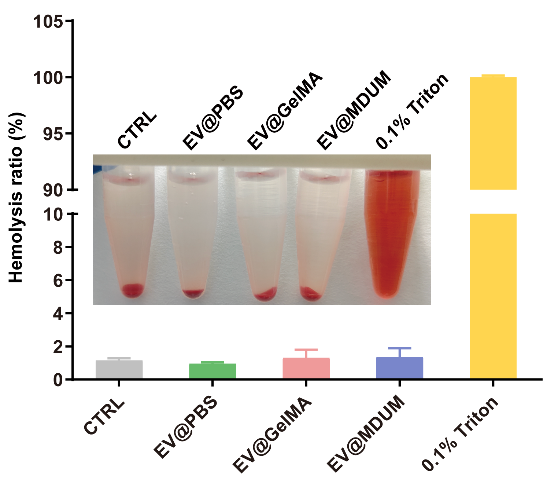


Fig. S1 Biocompatibility evaluation of EV@MDUM. The hemolysis rate of different groups. The photo showed the visualization of the hemolysis experiments after centrifugation, where the sediment at the bottom of the tubes is red blood cells.


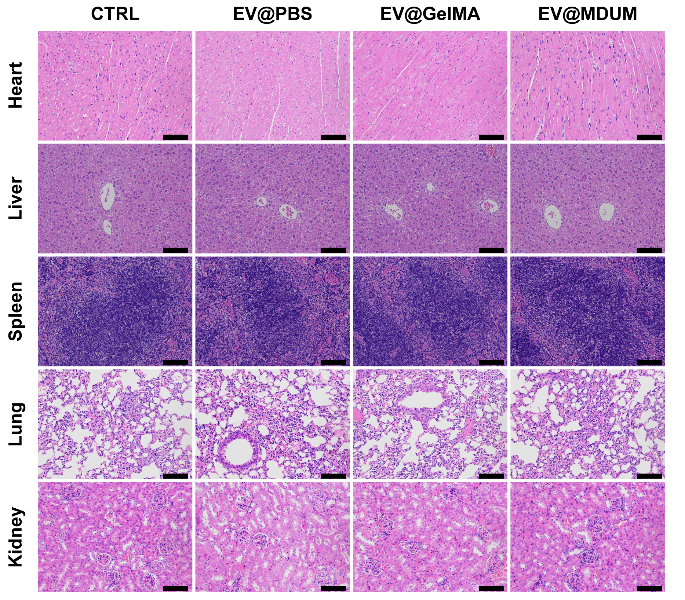


Fig. S2 Histological evaluation of major organs following EV@MDUM treatment in diabetic mice. Representative H&E staining images of the heart, liver, spleen, lung, and kidney harvested from diabetic mice treated with CTRL, EV@PBS, EV@GelMA, or EV@MDUM at the end of the in vivo study. Scale bar =100 μm.

Tables S1

**Supplementary Table 1 Primers sequences for real-time qPCR analysis**

| miRNA | Primer Sequences (5’-3’) |
| --- | --- |
| miRNA-126-3p | CTCGTACCGTGAGTAATAATGCG |
| miRNA-139-5p | TCTACAGTGCACGTGTCTCCAGT |
| miRNA-146a-5p | CTGAGAACTGAATTCCATGGGTT |
| miRNA-223-5p | CCGTGTATTTGACAAGCTGAGTT |
| cel-miR-39-3p | CGGGTGTAAATCAGCTTGAA |
