# Supplementary material for: A 3-Dimensional Bioprinted Decellularized Umbilical Cord Matrix Patch for Enhanced Storage and Delivery of Extracellular Vesicles in Diabetic Wound Healing
Source: Research (Wash D C). 2026 Apr 22;9:1246. doi: 10.34133/research.1246 (PMC13100348; doi:10.34133/research.1246)
Supplement: Supplementary 1 — Figs. S1 and S2 Table S1 [file research.1246.f1.zip › Tables S1.docx]

Tables S1

**Supplementary Table 1 Primers sequences for real-time qPCR analysis**

| miRNA | Primer Sequences (5’-3’) |
| --- | --- |
| miRNA-126-3p | CTCGTACCGTGAGTAATAATGCG |
| miRNA-139-5p | TCTACAGTGCACGTGTCTCCAGT |
| miRNA-146a-5p | CTGAGAACTGAATTCCATGGGTT |
| miRNA-223-5p | CCGTGTATTTGACAAGCTGAGTT |
| cel-miR-39-3p | CGGGTGTAAATCAGCTTGAA |
