# Supplementary figures and images for: A 3-Dimensional Bioprinted Decellularized Umbilical Cord Matrix Patch for Enhanced Storage and Delivery of Extracellular Vesicles in Diabetic Wound Healing
Source: Research (Wash D C). 2026 Apr 22;9:1246. doi: 10.34133/research.1246 (PMC13100348; doi:10.34133/research.1246)

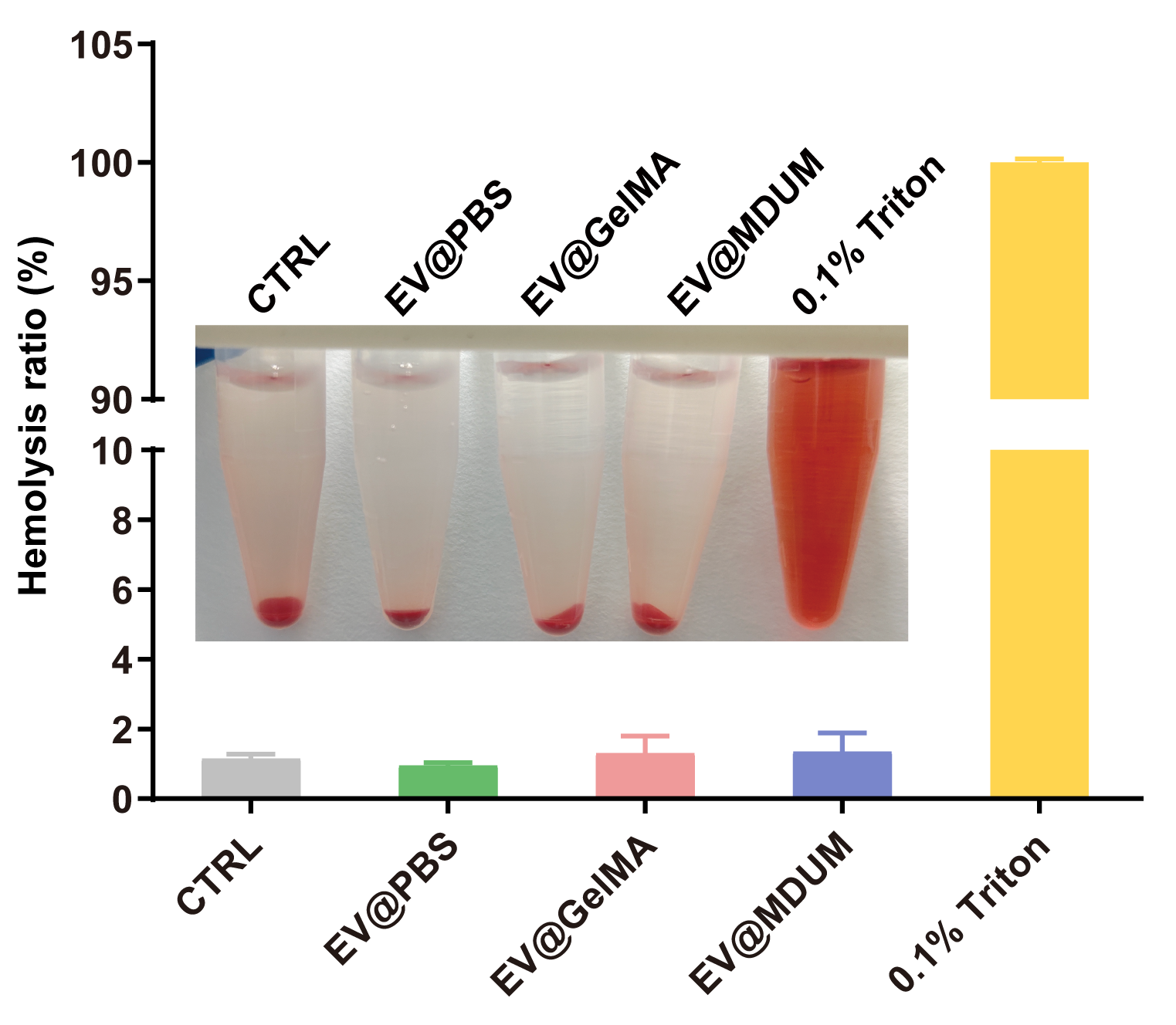

Supplement: Supplementary 1 — Figs. S1 and S2 Table S1 [file research.1246.f1.zip › supplementary data 1.png]

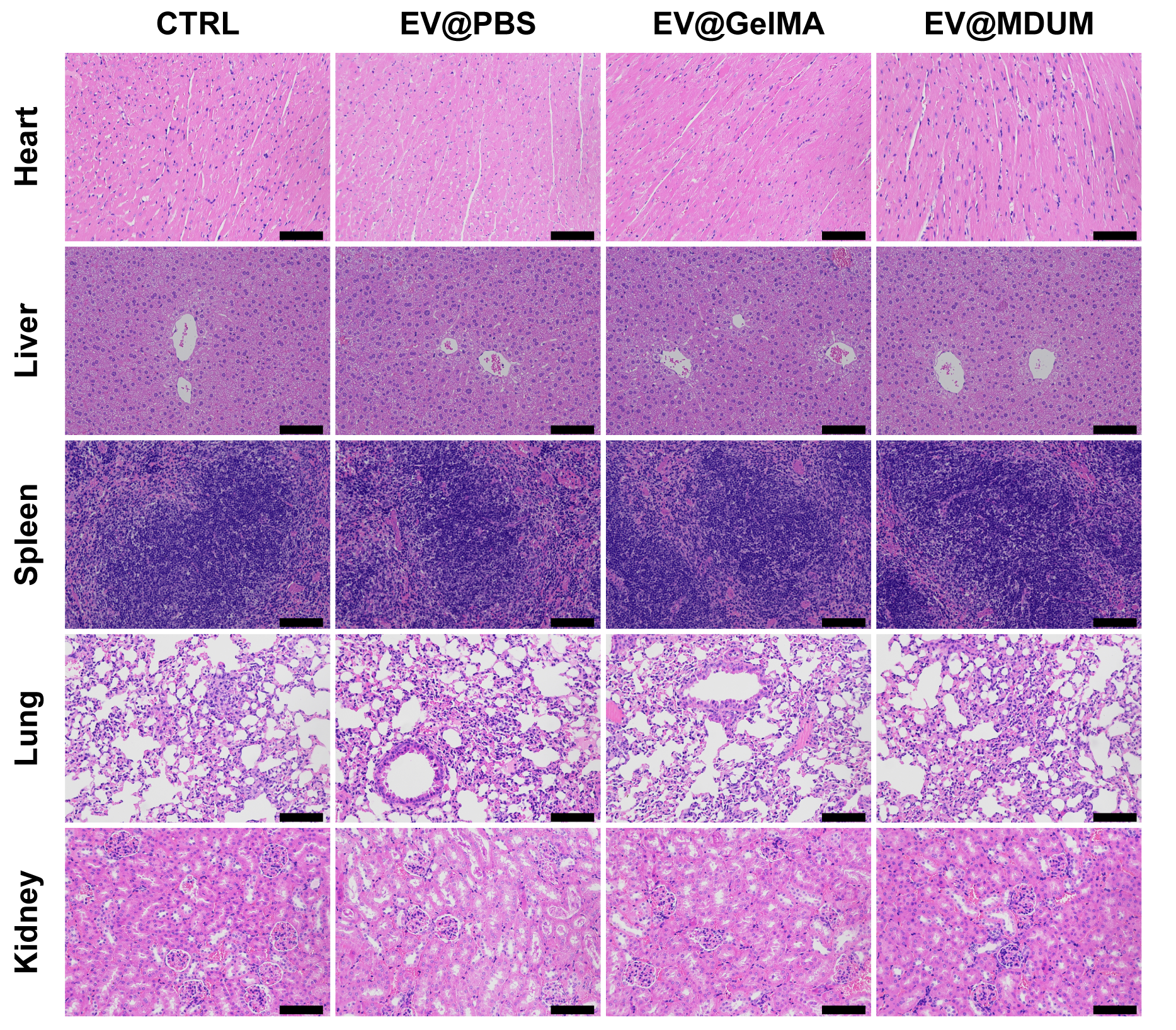

Supplement: Supplementary 1 — Figs. S1 and S2 Table S1 [file research.1246.f1.zip › supplementary data 2.png]
